# Valvular involvement in ANCA-associated systemic vasculitis: a case report and literature review

**DOI:** 10.1186/1471-2474-12-50

**Published:** 2011-02-23

**Authors:** Chloé Lacoste, Nicolas Mansencal, Mona Ben m'rad, Catherine Goulon-Goeau, Pascal Cohen, Loïc Guillevin, Thomas Hanslik

**Affiliations:** 1 Assistance Publique Hôpitaux de Paris (AP-HP), departments of internal medicine and Cardiology, Ambroise Paré Hospital, 9, avenue Charles-de-Gaulle, 92100, Boulogne Billancourt, France; 2 Université Versailles Saint Quentin en Yvelines, UFR de Médecine "Paris Ile-de-France Ouest", 9 boulevard d'Alembert, 78280 Guyancourt, France; 3 Assistance Publique Hôpitaux de Paris (AP-HP), National Referral Center for Rare Systemic and Autoimmune Diseases, Necrotizing Vasculitides, and Systemic Sclerosis, Department of Internal Medicine, Cochin Hospital, 27, rue du Faubourg Saint-Jacques, 75014, Paris, France; 4 Université Paris Descartes, 12, rue de l'Ecole de médecine 75006 Paris, France

## Abstract

**Background:**

Antineutrophil cytoplasmic antibodies (ANCA)-associated systemic vasculitides have a variety of presentations, but cardiac valvular involvement is rarely diagnosed and its management is not established.

**Case presentation:**

We report the case of a 44 year old man who presented with an ANCA-associated systemic vasculitis and aortic regurgitation of unusual mechanism. Transthoracic and transesophageal echocardiography disclosed septal hypertrophy preventing a complete closure of the aortic valve and thus responsible for a massive aortic regurgitation. After 4 months of immunosuppressive therapy, the valve lesion did not subside and the patient had to undergo aortic valve replacement. This report also reviews the 20 cases of systemic ANCA-associated vasculitis with endocardial valvular involvement previously reported in the English language medical literature.

**Conclusions:**

Valvular involvement in ANCA-associated systemic vasculitides is rarely reported. Most of these lesions are due to Wegener's granulomatosis and half are present when the diagnosis of vasculitis is made. The valvular lesion is usually isolated, aortic regurgitation being the most frequent type, and often requires valve replacement in the months that follow it's discovery.

## Background

Antineutrophil cytoplasmic antibodies (ANCA)-associated systemic vasculitides are a group of small vessel vasculitic syndromes including Wegener's granulomatosis, microscopic polyangiitis and Churg Strauss syndrome. These diseases share a common pathology with focal necrotizing lesions that affect different vessels and organs. Wegener's granulomatosis and Churg Strauss syndrome have additional granulomatous lesions. They have a variety of presentations, but cardiac valvular involvement is rarely reported.

In this report we describe the case of a patient with an ANCA associated systemic vasculitis who presented with aortic regurgitation of unusual mechanism requiring surgical replacement.

## Case report

The patient was a 44-year-old man from Bangladesh who immigrated to France twenty years ago and worked in several dusty embroidery workshops. Eight months prior to his admission in our hospital, he developed right chronic headaches resisting usual painkillers with right hypoacusia and tinnitus. The right ear examination was consistent with chronic otitis media. A sinus CT scan showed pansinusitis predominating on the right side with right mastoiditis and otitis media. A cerebral MRI showed pachymeningitis of the cerebellopontine angle. Right myringotomy yielded culture-negative drainage. The patient underwent several unsuccessful antibiotic treatments.

Eight months after the onset of the disease, the patient was referred to our ward, complaining, in addition to the other symptoms, of signs of severe intracranial hypertension (persistant right headaches and vomiting), significant weight loss, and a change in his voice. Otorhinological examination showed an unchanged right ear, a paralysis of the left vocal cord responsible for the dysphonia, an abolition of the gag reflex, and a palatal paralysis. The blood pressure was 140/65 mmHg, the heart rate of 75 bpm and the general examination was normal aside from a three out of four diastolic murmur of aortic regurgitation, without any other cardiac signs and a normal electrocardiogram.

**Figure 1:**
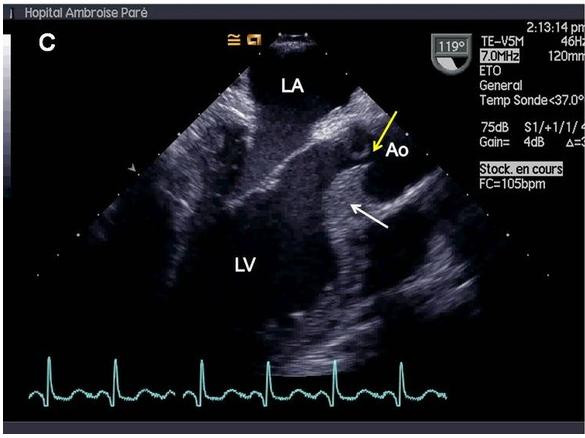
**Transesophageal echocardiography showing septal hypertrophy (white arrows) and lack of central coaptation of aortic valve (yellow arrows), resulting in massive aortic regurgitation**. Ao = aorta. LA = left atrium. LV = left ventricle.

An abdominal CT-scan showed a focal thickening of the aortic arch and an identical thickening of the superior mesenteric vein, interpreted as focal aortic and mesenteric vasculitis. A mesenteric panniculitis was also apparent. Laboratory studies included the following values: C-reactive protein 35 mg/l, serum urea nitrogen 4.6 mM/l, creatinine 86 mcM/l, total leukocyte count 10.3 × 10^9/l, polyclonal hypergammaglobulinemia, negative antiphospholipid antibodies, positive antineutrophil cytoplasmic antibodies (ANCA) with positive antimyeloperoxidase antibodies and negative antiproteinase 3 antibodies. The cerebrospinal fluid contained 60 cells per mm3 with 28% lymphocytes and 1.11 g/l of protein; the bacterial, fungal and mycobacterial cultures were negative. The cerebral MRI performed on admission was unchanged. Inferior nasal concha biopsies showed nonspecific inflammation; temporal artery biopsies were normal. Transthoracic and transesophageal echocardiography revealed septal hypertrophy leading to a restriction of aortic valvular closure and thus to massive aortic regurgitation (Figure 1).

The presumptive diagnosis was ANCA-associated systemic vasculitis, most probably Wegener's granulomatosis, with aortic valvular involvement. Treatment with prednisone (1 mg/kg) and intravenous cyclophosphamide (700 mg/m2 every two weeks during one month then every four weeks) was started. With this immunosuppressive therapy the palatal paralysis subsided, the headaches lessened and the patient, slowly regaining weight and energy, was able to return home.

Four months after the beginning of treatment there was an absence of echocardiographic improvement of the septal hypertrophy and aortic regurgitation and the patient had developed congestive heart failure, so the decision was made to perform an aortic Bicarbon™valve replacement. Upon inspection, the aorta wall and the aortic valve leaflets appeared diffusely thickened. There was a focal thickening of the interventricular septum covered with fibrous tissue. Fibrous tissue was also found on the aortic ring and on the large mitral leaflet. No vegetations were seen. At the end of surgery the patient was in persistent complete heart block requiring a pacemaker implantation.

The microscopic examination of the septum showed chronic remodelling with fibrous and hyaline tissue, and an intense polymorphic inflammatory infiltrate. Fibrosis as well as a local fragmentation of the elastic fibers was found in the intima and media of the aortic wall. No inflammatory infiltrate nor granuloma were seen in the aortic specimens.

Two years after surgery, the patient is doing well on azathioprine (see Table 1).

**Table 1:** Symptoms, severity score index and main lab and imagery results of the patient described in the case report.

November 2006	- Persisting facial pain, right chronic headaches resisting usual pain killers
	- Cerebral CT-scan: pansinusitis
March 2007	- Same symptoms + right hypoacusia and tinnitus
	- Cerebral CT-scan: pansinusitis predominating on the right side with right mastoiditis and otitis media
	- Cerebral MRI: pachymeningitis of the cerebellopontine angle

June 2007	When hospitalized:
	- Violent headaches and vomiting: severe intracranial hypertension
	- Significant weight loss (- 10 kg)
	- Right otitis media
	- Paralysis of the left vocal cord (dysphonia), abolition of the gag reflex, and palatal paralysis
	- Cerebral MRI: unchanged
	- Spinal tap: Aseptic meningitis
	- Discovery of the aortic regurgitation
	- Abdominal CT-scan: focal aortic and mesenteric vasculitis and mesenteric panniculitis
	- Elevated C-reactive protein and leukocyte count
	- Polyclonal hypergammaglobulinemia
	- pANCA+; anti-MPO +; anti Pr3 -
	- BVAS = 23

June 2009	BVAS = 2

BVAS: Birmingham Vasculitis Activity Score

## Discussion

In this patient, the diagnosis of ANCA-associated systemic vasculitis, most probably Wegener's granulomatosis, was based on the association of pansinusitis, otitis, mastoiditis, chronic lymphocytic meningitis, aortitis and positive ANCA. This diagnosis was assessed at the National Referral Center for Rare Systemic and Autoimmune Diseases, Necrotizing Vasculitides, and Systemic Sclerosis (Hôpital Cochin, Assistance Publique-Hôpitaux de Paris, Université Paris-Descartes, Paris). Although Pr3-ANCA, found in 70 to 90% of patients with Wegener's granulomatosis, is often considered to be a seromarker of this disease, MPO-ANCA has been reported to be predominant in Asian patients [1]. This could explain the positive p-ANCA and negative c-ANCA in this patient who is from Bangladesh. The very unusual and unique feature in this patient was the particular mechanism of aortic valve involvement that required a valve replacement.

Using a Pubmed search we looked for reported cases of ANCA-associated systemic vasculitis with a valvular involvement, using the keywords "valve", "cardiac", "heart", "endocarditis", "mitral", "aortic", "tricuspid", "pulmonary", "ANCA", "antineutrophil cytoplasmic antibodies", "Wegener's granulomatosis", "microscopic polyangiitis" and "Churg Strauss" in different combinations. We completed this search by cross-referencing published articles. We thus selected 19 articles in English reporting 20 cases consistent with a systemic ANCA-associated vasculitis with endocardial valvular involvement, i.e. valvular lesions for which infective endocarditis had been excluded and no other aetiology found [2-20].

The general characteristics of the patients and of their vasculitides are reviewed in Table 2. Their average age tends to be younger than that of the other patients presenting an ANCA-associated-vasculitis (40 versus 47 years old [21]) and the male predominance is greater (80% versus 53% [21]). One case excepted [19], all reported cases were diagnosed as Wegener's granulomatosis and presented with at least one of the three most common involvements, i.e. renal, pulmonary and otolaryngological. Eye, skin or joint involvement was present in about half the cases. Only 6 patients out of 20 experienced another cardiac lesion, such as pericardial effusion or adhesions [6,10,15], conduction disorders [4,9,10] and coronary artery stenosis [12].

**Table 2:** General characteristics of patients with systemic vasculitides and valvular involvement.

Reference	Sex	Age	ANCA	Vasculitis Classification	Kidney	ENT	Organ Lungs	Involvement Eyes	Skin	Joints	Non-valvular cardiac lesion
Stöllberger [19]	M	56	ANCA -, anti-PR3 +	Unknown	Yes	-	-	-	Yes	Yes	-

Levine [15]	M	28	**-**	Wegener's granulomatosis	Yes	Yes	Yes	-	-	-	Yes

Davenport [6]	M	19	c-ANCA +, anti-PR3 +	Wegener's granulomatosis	Yes	Yes	Yes	Yes	Yes	Yes	-
	
	M	53	c-ANCA +, anti-PR3 +	Wegener's granulomatosis	Yes	-	-	-	-	-	Yes

Grant [10]	M	32	c-ANCA +	Wegener's granulomatosis	-	Yes	-	Yes	Yes	Yes	Yes

Goodfield [9]	M	25	c-ANCA +	Wegener's granulomatosis	-	Yes	Yes	-	-	-	Yes

Bruno [4]	F	63	ANCA +	Wegener's granulomatosis	-	Yes	Yes	-	-	-	Yes

Herbst [12]	F	56	ANCA -	Wegener's granulomatosis	-	-	Yes	-	-	Yes	Yes

Gerbracht [8]	M	20	-	Wegener's granulomatosis	Yes	Yes	Yes	-	-	-	-

Greidinger [11]	M	15	c-ANCA +	Wegener's granulomatosis	Yes	Yes	Yes	-	Yes	Yes	-

Leff [14]	M	17	c-ANCA +	Wegener's granulomatosis	-	Yes	Yes	-	Yes	Yes	-

Yanda [20]	F	77	-	Wegener's granulomatosis	Yes	Yes	Yes	Yes	Yes	-	-

Dabbagh [5]	M	16	-	Wegener's granulomatosis	Yes	Yes	Yes	Yes	-	-	-

Fox [7]	M	20	ANCA +	Wegener's granulomatosis	Yes	Yes	Yes	Yes	Yes	Yes	-

Anthony [2]	M	48	c-ANCA +, anti-PR3 +	Wegener's granulomatosis	-	Yes	Yes	-	Yes	Yes	-

Paik [17]	M	48	c-ANCA +	Wegener's granulomatosis	-	Yes	Yes	-	-	-	-

Mishell [16]	M	65	ANCA +, anti-PR3 +	Wegener's granulomatosis	Yes	-	Yes	Yes	Yes	-	-

Ramakrishnan [18]	F	44	c-ANCA +	Wegener's granulomatosis	Yes	-	Yes	-	-	-	-

Attaran [3]	M	52	-	Wegener's granulomatosis	-	Yes	-	-	-	-	-

Koyalakonda [13]	M	52	-	Wegener's granulomatosis	-	Yes	-	Yes	-	-	-

Present report	M	44	p-ANCA +, anti-PR3 -	Wegener's granulomatosis	-	Yes	-	-	-	-	-

ANCA: antineutrophil cytoplasmic antibodies; ENT: ear, nose and/or throat involvement; PR3: proteinase-3.

Table 3 details the characteristics of the valvular lesions in the 20 different cases. The most commonly encountered disorder is aortic regurgitation [4-11,13,14,19,20]. Seven cases of mitral regurgitation [3,7,11-13,15,20], and one of aortic stenosis [2] were also reported along with two cases of valvular vegetations [16,17], two of a mass involving a mitral leaflet responsible for mild mitral stenosis and moderate mitral regurgitation [3,13], and one of multiple atrial masses without valvular insufficiency or stenosis [18]. Six patients had polyvalvular involvement [3,7,11,13,16,20]. Several mechanisms responsible for these valvular lesions have been reported: vegetations [2,8,16,17,19], leaflet thickening [4,6,9,13,20], valvular perforation [6,14] and unique or multiple endocardial masses [3,9,12,13,18].

**Table 3:** Valvular lesion characteristics in patients with systemic vasculitides.

Reference	Valve lesion onset	Valve lesion	Mechanism of valvular disease	Valvular treatment	Outcome	Valve histology
Stöllberger [19]	After 3 days steroïds	AR	Vegetation	-	AVR	Non specific
Levine [15]	After 6 weeks of ENT signs no IST	MR	?	-	Died of heart failure	-
Davenport [6]	At initial presentation	AR	Leaflet perforation and tissue disruption	1 year IST	AVR	Non specific
	At initial presentation	AR	Thickened leaflets	IST	Worse, awaiting AVR	-
Grant [10]	After 1 year of CYC	AR	Dilation of ascending aorta	20 Mo IST	AVR	Non specific
Goodfield [9]	At initial presentation	AR	Thickened leaflets and mass obstructing the left ventricular outflow tract	6 weeks IST	Thickening & mass disappeared. AVR because of LV dilatation & shrunken Ao leaflet	Non specific
Bruno [4]	3 years after illness onset, no IST	AR	Thickened, rigid and retracted leaflets	_	AVR	Specific
Herbst [12]	At initial presentation	MR	Mass involving a leaflet		MVR & AVR	Specific
Gerbracht [8]	After 5 days CYC	AR	Vegetation	IST	Complete resolution	-
Greidinger [11]	After 3 weeks CYC	AR	?	IST	Lesion unchanged	-
		MR	?	IST	Lesion unchanged	-
Leff [14]	At initial presentation	AR	Perforation of 2 leaflets	1 year IST	Ao valve repair: homograft	-
Yanda [20]	1 year after initial presentation, no IST	AR	Thickened leaflet	-	AVR	Non specific
		MR	Thickened leaflet	IST	?	-
Dabbagh [5]	After 3 weeks CYC	AR	?	IST	?	-
Fox [7]	At initial presentation	AR	Prolapsing Ao leaflets and discrete Ao leaflet deficiency	5 Mo IST	AVR	Non specific
		MR	?	IST	?	-
Anthony [2]	At initial presentation	AS	Vegetation	3 Mo IST	Lesion unchanged	-
Paik [17]	At initial presentation	Ao vegetations	Vegetation	IST	?	
Mishell [16]	At initial presentation	Ao and M vegetations	Vegetations	IST	Died	Non specific
Ramakrishnan [18]	At initial presentation	M masses	Multiple masses in atriums and on MP	Few days IST	Died	-
Attaran [3]	30 years after illness onset, IST?	Ao and M mass, MR, MS	Mass involving an Ao and an M leaflet	IST	MVR & AVR	Non specific
Koyalakonda [13]	Not at initial presentation and under treatment	MS & MR AR	M mass involving leaflet Thickened Ao valve	IST IST	Mass unchanged: MVR AR progressed: AVR	Non specific
Present report	At initial presentation	AR	Septal thickening: incomplete closure of Ao valve, thickened leaflets	4 Mo IST	AVR	Non specific

?: Unknown; AR: aortic regurgitation; Ao: aortic; AS: aortic stenosis; AVR: aortic valve replacement; CYC: cyclophosphamide; IST: immunosuppressive therapy; LV: left ventricular; M: mitral; Mo: months; MP: mitral prothesis; MR: mitral regurgitation; MS: mitral stenosis; MVR: mitral valve replacement.

Half the valvular lesions were present at initial presentation and when the diagnosis of vasculitis was made [2,6,7,9,12,14,16-18]. In six of the ten remaining cases, the valvular disease occurred while the patient was on immunosuppressant drugs [5,8,10,11,13,19]. In two of these five cases, the onset was less than a week after the treatment was initiated suggesting that the pathological process was already taking place when the drugs were started [8,19]. In only four cases valvular disease began more than a year after the first signs of the vasculitis [3,4,10,20].

A valve replacement was required in most cases [3,4,6,7,9,10,12,13,19,20]. In only two cases did immunosuppressive therapy allow a complete resolution of the valvular lesions [8,9]; and in one of these [9] the patient had nonetheless to undergo an aortic valve replacement because of secondary left ventricular dilatation and a shrunken leaflet.

The pathological findings were nonspecific in almost all cases; i.e. showing inflammation and/or scarring without abscess, giant cells, vasculitis, or granuloma. In two cases, granuloma, necrosis and/or micro abscess were described in the valvular tissue [4,12].

To conclude, although it might be underdiagnosed due to the lack of patent clinical signs and the absence of systematic screening, valvular involvement in ANCA-associated systemic vasculitides is rarely reported. Most of these valvular lesions are due to Wegener's granulomatosis and half are present when the diagnosis of vasculitis is made. The valvular lesion is usually unique, aortic regurgitation being the most frequent type, and often requires valve replacement in the months that follow its discovery.

## Consent

written informed consent was obtained from the patient for publication of this case report and any accompanying images.

## Competing interests

The authors declare that they have no competing interests.

## Authors' contributions

CL wrote the paper; NM conducted the cardiological expertise; CGG, conducted the neurological expertise; MB,.PC and LG conducted the patient's evaluation at the National Referral Center for Rare Systemic and Autoimmune Diseases, Necrotizing Vasculitides, and Systemic Sclerosis (Hôpital Cochin, Assistance Publique-Hôpitaux de Paris, Université Paris-Descartes, Paris); TH is taking care of this patient and supervised the writing of this paper. All the authors have made substantial contributions to acquisition and analysis of data and have been involved in revising the manuscript critically for intellectual content.

All authors read and approved the final manuscript.

## Pre-publication history

The pre-publication history for this paper can be accessed here:

http://www.biomedcentral.com/1471-2474/12/50/prepub
